# Complicated Appendicitis in a Pediatric Patient With COVID-19: A Case Report

**DOI:** 10.7759/cureus.8677

**Published:** 2020-06-17

**Authors:** Abdulaziz B Alsuwailem, Reem Turkistani, Mashael Alomari

**Affiliations:** 1 Emergency Department, King Abdulaziz Medical City, Ministry of National Guard Health Affairs, Riyadh, SAU; 2 Pediatric Emergency Medicine, King Abdulaziz Medical City, Ministry of National Guard Health Affairs, Riyadh, SAU

**Keywords:** appendicitis, covid-19, pediatric

## Abstract

Perforated appendicitis is a well-known complication of acute appendicitis (AA), which increases the morbidity rate in children. This report discusses the case of a pediatric patient with perforated appendicitis with a secondary diagnosis of coronavirus disease 2019 (COVID-19). The clinical presentation of complicated appendicitis (CA) in association with COVID-19 may not be different from that in the general population. Our patient underwent successful management with open appendectomy and subsequent antibiotics. There is no evidence to alter the standard of care for such patients. So further studies are needed to provide more clarity on the appropriate management.

## Introduction

Acute appendicitis (AA) is one of the most commonly reported causes of acute abdomen in children presenting to the ED. Yet, it is difficult to differentiate it from other causes as less than 50% of patients present with classic symptoms [1]. Classically, they present with low-grade fever, malaise, and anorexia and then progress to abdominal pain and vomiting. Diagnosis is usually based on clinical examination; however, other modes of investigations such as ultrasound and CT are helpful in some difficult cases [2]. A delayed or missed diagnosis can result in major complications such as appendiceal perforation.

The clinical presentation of AA in association with a positive coronavirus disease 2019 (COVID-19) test in children has not been previously reported in the literature during the current COVID-19 pandemic outbreak period. Here, we present the case of a four-year-old girl who presented to the ED with complicated appendicitis (CA) and tested positive for COVID-19.

## Case presentation

A four-year-old girl presented to the ED with a history of progressive, severe, and generalized abdominal pain that had started three days prior. The pain was associated with a subjective fever responsive to antipyretic (acetaminophen) given at home and non-bloody, non-bilious vomiting. Her family reported a decrease in oral intake and hypoactivity and no change in bowel habits, runny nose, throat pain, cough, cyanosis, or shortness of breath. No urinary tract or neurological symptoms were present. The family also reported a history of direct contact with one of her relatives at home who had symptoms of upper respiratory tract infection but was not tested for COVID-19. Her past medical history was unremarkable for any chronic illnesses or previous surgical interventions.

The patient was examined after wearing personal protective equipment, and physical examination revealed an adequately growing, well-nourished, ill-looking child. She had a body temperature of 39.1 °C, respiratory rate of 60, a pulse of 168 beats/min, blood pressure of 118/77 mmHg, and oxygen saturation of 100% on room air. Her abdomen was tense and rigid with diffuse tenderness. Significant rebound tenderness was observed. Chest examination showed that her lung sounds were normal with equal air entry on both sides.

**Figure 1:**
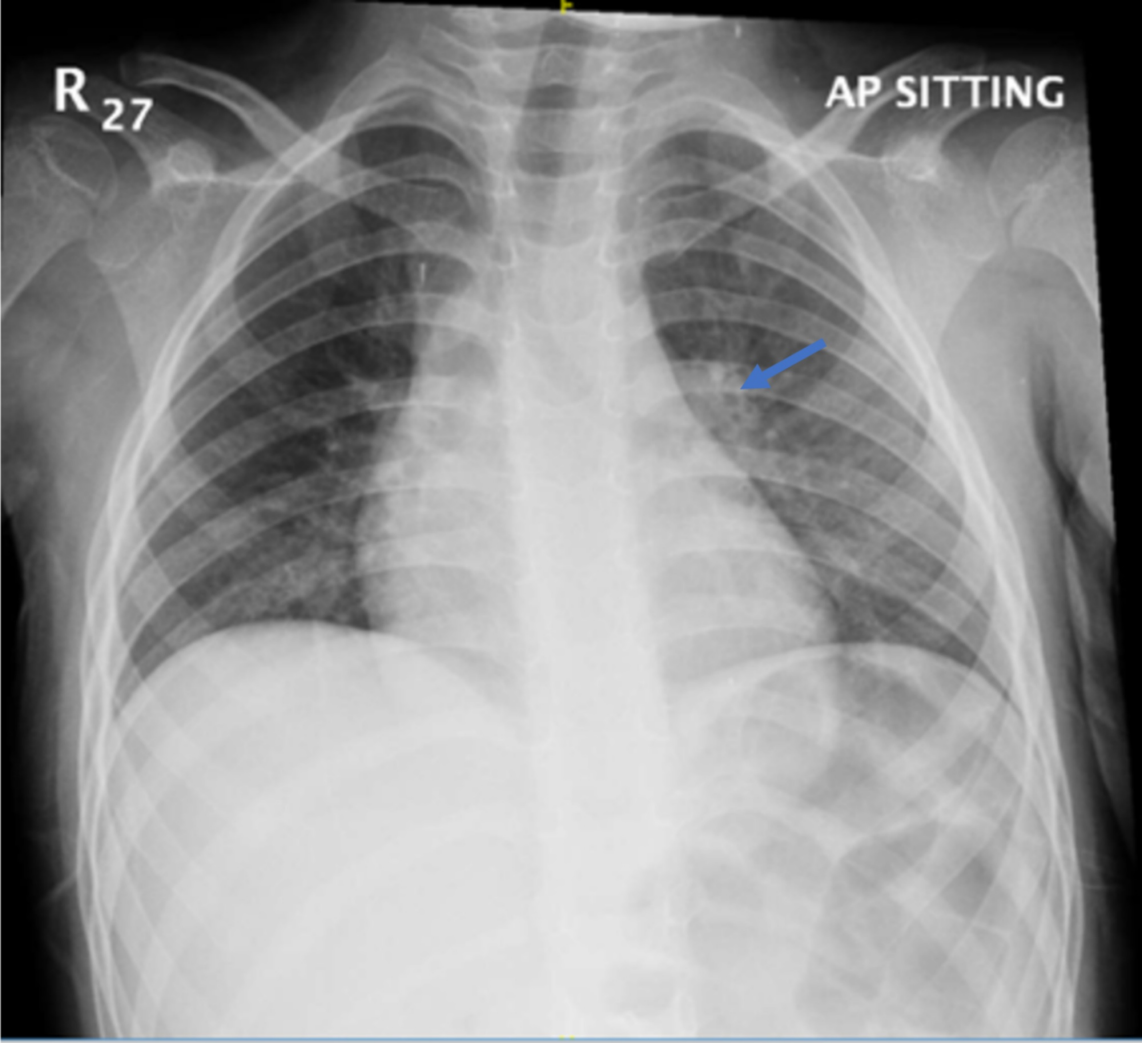
Chest X-ray of the patient The arrow demonstrates peri-bronchial wall thickening indicating small airway disease

On initial laboratory workup, the complete blood count showed a slight elevation in white blood cell count of 12.3 × 109 cells/L and neutrophil count of 10 × 109 cells/L; all other investigations were normal including liver function tests, amylase, lipase, electrolytes, and creatinine. Routine chest X-ray was performed and showed bilateral peri-bronchial wall thickening indicating small airway disease as demonstrated in Figure 1.

**Figure 2:**
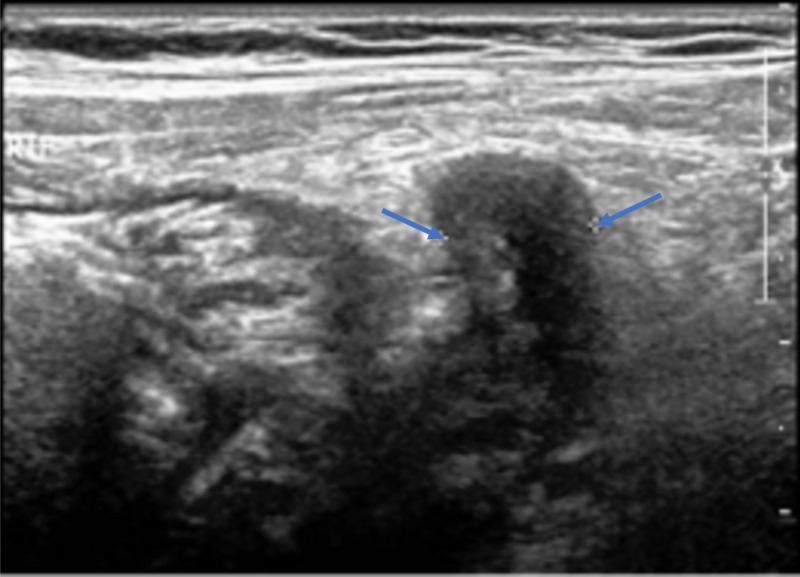
Ultrasonography of the abdomen - view 1 The image shows noncompressibility and discontinuity in the appendicular wall, with a maximum diameter of 0.9 cm (arrows)

**Figure 3:**
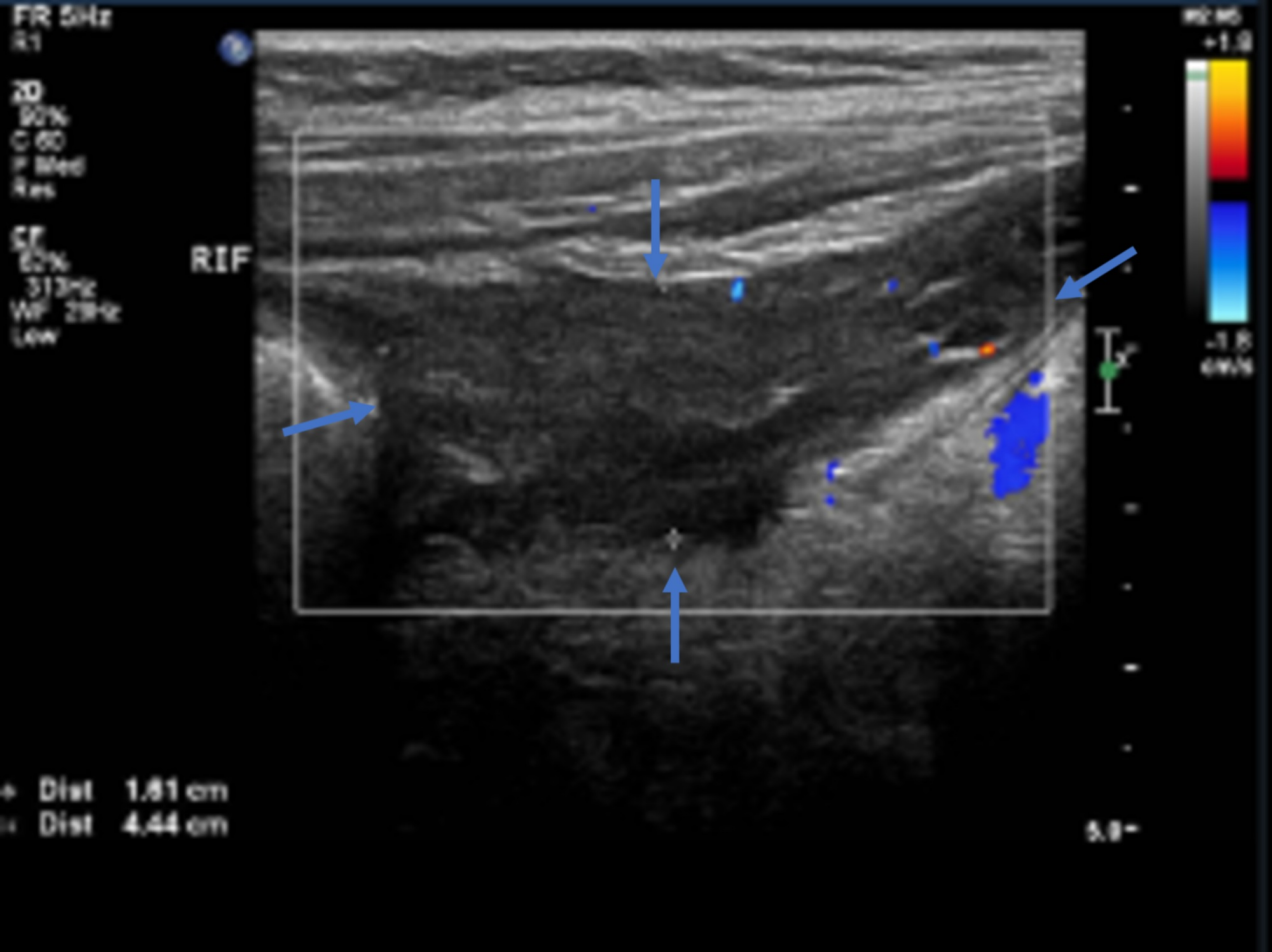
Ultrasonography of the abdomen - view 2 The image shows adjacent turbid collection (arrows) measuring 1.6 × 4.4 cm, indicating perforated appendicitis

Ultrasonography of the abdomen revealed noncompressibility and discontinuity in the appendicular wall (Figure 2), with adjacent turbid collection indicating perforated appendicitis (Figure 3).

Hence, a diagnosis of perforated appendicitis was confirmed at that time. After initial stabilization in the ED with a bolus of normal saline, intravenous antibiotics including ceftriaxone, metronidazole, and clindamycin were administered. The patient was tested for COVID-19 with polymerase chain reaction (PCR) for severe acute respiratory syndrome coronavirus 2 (SARS-CoV-2) from the nasopharyngeal swab, as this is the admission protocol in our institution during the current COVID-19 outbreak. She was taken to the operating room for an emergency open appendectomy. Intraoperatively, after opening the peritoneum, gushing pus was removed. Further dissection was done until the appendix was visualized and found to be gangrenous with a healthy base; the appendix was subsequently removed. The patient tolerated the surgery well with no immediate postoperative complications. On the first postoperative day, her SARS-CoV-2 PCR test result came back positive. The patient was placed in a regular surgical ward and isolated in a negative pressure room. She received a course of intravenous antibiotics including ceftriaxone and metronidazole for five days followed by a shift to oral amoxicillin/clavulanic acid for seven days. The patient experienced a smooth hospital stay of 12 days and was discharged in a stable condition, with instructions for self-isolation at home. Education was provided regarding COVID-19, its mode of transmission, precautions to be be taken, and the importance of hand hygiene and social distancing.

## Discussion

AA is a common surgical disease in children. The diagnosis can be difficult and delayed in some cases due to atypical clinical presentation, lack of an accurate and detailed history, and poor cooperation with the physical examination, all of which can lead to misdiagnosis. Moreover, its presentation can sometimes mimic other conditions such as gastroenteritis [3]. In our patient, the medical history and physical examination were suggestive of CA. Delay in diagnosis is common in young children and has been reported in 57% of cases involving children less than six years of age [4]. It correlates with an increased risk of significant morbidity such as appendicular perforation, thereby increasing the length of hospital stay [5]. In our case, perforated appendicitis was suspected as the patient had been suffering from progressive and severe abdominal pain for three days prior to ED visit; moreover, abdominal rigidity was found upon physical examination.

In a retrospective study, Gonzalez et al. found that using the ultrasound as an imaging modality to diagnose AA is sufficient and may help confirm the diagnosis, especially in CA, as its specificity is greater than 87% [6]. In a report of eight children with COVID-19 who presented to a single center in the United Kingdom with gastrointestinal symptoms suspected to be associated with atypical appendicitis, they emphasized the importance of visualizing the appendix through ultrasound, CT, or both, as imaging was the only diagnostic modality to exclude appendicitis in the reported cases [7]. In our case, using the departmental ultrasound was an excellent decision to confirm the diagnosis of CA. Thus, exposure to abdominal CT radiation was avoided. Our patient was assessed by a pediatric surgeon and the decision to perform an emergent open appendectomy was made as she looked ill and her ultrasound showed significant turbid collection. In a meta-analysis of 14 studies, Fugazzola et al. compared the non-operative management (NOM) of CA with operative management (OM) and showed that complication rate, re-admission, and cost were the same between the two groups [8]. However, the length of hospital stay was decreased with OM, although the difference was not statistically significant.

Since the COVID-19 outbreak, there have only been two reported cases of acute non-CA with positive COVID-19 PCR test results in adults. The first reported case was of an adult with multiple comorbidities complaining of abdominal pain and dyspnea. He was treated medically with piperacillin/tazobactam for appendicitis. The decision to treat with NOM was made because of the high operative risk to the patient, as well as the risk of exposing the healthcare providers to COVID-19. The patient was admitted to a regular ward and received hydroxychloroquine and azithromycin to treat COVID-19 [9]. The other case involved a healthy adult patient who had a classic presentation of AA, which was confirmed by CT. The patient was treated surgically with no evidence of COVID-19 detected in the peritoneal fluid by PCR [10].

There is limited data in the literature regarding such cases in the pediatric population. Our patient did not receive hydroxychloroquine or azithromycin or any other agent for her COVID-19 infection because she had no respiratory symptoms and could breathe well in room air. Thus, this case study shows successful treatment with open appendectomy for CA in a child with COVID-19 with good recovery and no sequelae.

## Conclusions

The clinical presentation of CA in association with COVID-19 may not be different from that in the general population. Our patient underwent successful management with open appendectomy with subsequent antibiotics. There is no evidence for the need to alter the standard of care for patients with similar conditions. So further studies are needed to provide more clarity on the appropriate management.

## References

[1] (2020). Society of American Gastrointestinal and Endoscopic Surgeons: pediatric appendicitis. https://www.sages.org/wiki/pediatric-appendicitis/.

[2] Pade KH (2018). Point-of-care ultrasound facilitates bedside diagnosis of appendicitis with an appendicolith in a pediatric patient. Pediatr Emerg Care.

[3] Wang ZH, Ye J, Wang YS, Liu Y (2019). Diagnostic accuracy of pediatric atypical appendicitis: three case reports. Medicine (Baltimore).

[4] Rothrock SG, Pagane J (2000). Acute appendicitis in children: emergency department diagnosis and management. Ann Emerg Med.

[5] Meltzer JA, Kunkov S, Chao JH (2019). Association of delay in appendectomy with perforation in children with appendicitis. Pediatr Emerg Care.

[6] Gonzalez DO, Lawrence AE, Cooper JN (2018). Can ultrasound reliably identify complicated appendicitis in children?. J Surg Res.

[7] Tullie L, Ford K, Bisharat M (2020). Gastrointestinal features in children with COVID-19: an observation of varied presentation in eight children (Epub ahead of print). Lancet Child Adolesc Health.

[8] Fugazzola P, Coccolini F, Tomasoni M, Stella M, Ansaloni L (2019). Early appendectomy vs. conservative management in complicated acute appendicitis in children: a meta-analysis. J Pediatr Surg.

[9] Suwanwongse K, Shabarek N (2020). Successful Conservative management of acute appendicitis in a coronavirus disease 2019 (COVID-19) patient. Cureus.

[10] Ngaserin SH, Koh FH, Ong BC, Chew MH (2020). COVID-19 not detected in peritoneal fluid: a case of laparoscopic appendicectomy for acute appendicitis in a COVID-19-infected patient. Langenbecks Arch Surg.

